# The cAMP responsive element binding protein 1 transactivates epithelial membrane protein 2, a potential tumor suppressor in the urinary bladder urothelial carcinoma

**DOI:** 10.18632/oncotarget.3312

**Published:** 2015-04-13

**Authors:** Chien-Feng Li, Wen-Jeng Wu, Wen-Ren Wu, Yu-Jing Liao, Lih-Ren Chen, Chun-Nung Huang, Ching-Chia Li, Wei-Ming Li, Hsuan-Ying Huang, Yi-Ling Chen, Shih-Shin Liang, Nan-Haw Chow, Yow-Ling Shiue

**Affiliations:** ^1^ Department of Pathology, Chi Mei Medical Center, Tainan, Taiwan; ^2^ National Institute of Cancer Research, National Health Research Institutes, Tainan, Taiwan; ^3^ Department of Biotechnology, Southern Taiwan University of Science and Technology, Tainan, Taiwan; ^4^ Graduate Institute of Medicine, College of Medicine, Kaohsiung Medical University, Kaohsiung, Taiwan; ^5^ Department of Urology, School of Medicine, College of Medicine, Kaohsiung Medical University, Kaohsiung, Taiwan; ^6^ Department of Urology, Kaohsiung Medical University Hospital, Kaohsiung, Taiwan; ^7^ Department of Urology, Kaohsiung Municipal Hsiao-Kang Hospital, Kaohsiung, Taiwan; ^8^ Institute of Biomedical Sciences, National Sun Yat-sen University, Kaohsiung, Taiwan; ^9^ Division of Physiology, Livestock Research Institute, Tainan, Taiwan; ^10^ Department of Urology, Kaohsiung Municipal Ta-Tung Hospital, Kaohsiung, Taiwan; ^11^ Department of Urology, Ministry of Health and Welfare Pingtung Hospital, Pingtung, Taiwan; ^12^ Department of Pathology, Chang Gung Memorial Hospital, Kaohsiung Medical Center, Chang Gung University College of Medicine, Kaohsiung, Taiwan; ^13^ Department of Biotechnology, Kaohsiung Medical University, Kaohsiung, Taiwan; ^14^ Department of Pathology, National Cheng Kung University Hospital, Tainan, Taiwan; ^15^ Institute of Basic Medical Science, College of Medicine, National Cheng Kung University, Tainan, Taiwan; ^16^ Department of Biological Sciences, National Sun Yat-sen University, Kaohsiung, Taiwan; ^17^ Doctoral degree program in Marine Biotechnology, National Sun Yat-sen University, Kaohsiung, Taiwan

**Keywords:** EMP2, CREB1, urinary bladder urothelial carcinoma, tumor suppressor

## Abstract

In this study, we report that *EMP2* plays a tumor suppressor role by inducing G_2_/M cell cycle arrest, suppressing cell viability, proliferation, colony formation/anchorage-independent cell growth via regulation of G_2_/M checkpoints in distinct urinary bladder urothelial carcinoma (UBUC)-derived cell lines. Genistein treatment or exogenous expression of the cAMP responsive element binding protein 1 (*CREB1*) gene in different UBUC-derived cell lines induced *EMP2* transcription and subsequent translation. Mutagenesis on either or both cAMP-responsive element(s) dramatically decreased the *EMP2* promoter activity with, without genistein treatment or exogenous CREB1 expression, respectively. Significantly correlation between the EMP2 immunointensity and primary tumor, nodal status, histological grade, vascular invasion and mitotic activity was identified. Multivariate analysis further demonstrated that low EMP2 immunoexpression is an independent prognostic factor for poor disease-specific survival. Genistein treatments, knockdown of *EMP2* gene and double knockdown of *CREB1* and *EMP2* genes significantly inhibited tumor growth and notably downregulated CREB1 and EMP2 protein levels in the mice xenograft models. Therefore, genistein induced *CREB1* transcription, translation and upregulated pCREB1(S133) protein level. Afterward, pCREB1(S133) transactivated the tumor suppressor gene, *EMP2*, *in vitro* and *in vivo*. Our study identified a novel transcriptional target, which plays a tumor suppressor role, of CREB1.

## INTRODUCTION

Urinary bladder urothelial carcinoma (UBUC) is a common malignant disease with preferences for developed countries [1]. Environmental and genetic factors impact in its development [2–4]. Clinicopathological features including histological grade, stage, size and multiplicity are associated with its progression [5]. Despite improvements in surgical techniques and multimodal therapy, 5-year survival rates for patients with muscle-invasive UBUC remain suboptimal. Almost 50% of patients eventually progress and develop systemic disease [6]. Clinical and genetic heterogeneity observed in UBUC patients further complicates the use of general therapies [7]. One current and future strategy to improve existing treatment outcomes is to identify involving biological molecules for targeted therapies. Cell cycle dysregulation resulting in uncontrolled cell proliferation has been associated with UBUC development [8, 9]. Thus targeting a critical transcription factor to restore its function is a rational approach for UBUC treatments [10].

Genistein is believed to be a potent anticancer agent and has been shown to prevent carcinogenesis in animal models for tumor development at different organ sites [11]. Our previous study using suppression subtractive hybridization approach identified that genistein induced epithelial membrane protein 2 (*EMP2*) mRNA in UBUC-derived RT4 cells. High EMP2 immunointensity was recognized as a prognostic indicator for patients with upper tract urothelial carcinoma (UTUC), possibly via suppression of cell proliferation [12]. Relative to primary UBUC, UTUC is uncommon [13], with notable differences at the genetic, molecular and clinical levels [14–16]. Due to the functions of *EMP2* on UBUC and the underlying regulatory mechanisms remained elucidative, we performed data mining targeting the Gene Ontology (GO) with biological process of *cell proliferation* (GO:0008283) in the Gene Expression Omnibus (GEO, NCBI) database. Of 14 candidate transcripts, only downregulation of *EMP2* significantly predicts inferior overall survival (Supplementary Table S1, Figure S1), suggesting that *EMP2* plays a potential tumor suppressor role in UBUC.

Human *EMP2* mapped to chromosome 16, is highly conserved across vertebrates [17, 18]. The expression pattern of *EMP2* partially overlaps to that of the peripheral myelin protein 22 transcript (*PMP22*, also known as arrest-specific-3, *GAS3*). By containing the claudin domain and sharing approximately 40 amino acid identity with PMP22/GAS3 [19], EMP2 protein was detected as a novel member of this tetraspan transmembrane superfamily [20]. In humans, EMP2 protein has a discrete cell type and tissue distribution, with high levels observed in the lung and moderate levels in the eye, heart, thyroid and intestine [21, 22]. Results from our previous study [12], data mining and the fact that membrane proteins belong to the largest class of drug targets [23], prompted us to systematically analyze the relevance of EMP2 immunointensity and clinicopathological features in UBUC patients. Biological functions and a potential transcription factor of *EMP2* were also studied using three UBUC-derived cell lines, RT4, TSGH8301 and J82.

## RESULTS

### Data mining identified that *EMP2* transcript is frequently downregulated in high pT status patients with UBUC

To identify potential candidates related to the development of UBUC, we performed data mining. From the transcriptomic profiles of 93 UBUCs deposited in GEO dataset, 714 probes covering 317 transcripts which associated with the biological process of *cell proliferation* (GO:0008283) were found. The log_2_ ratios of 14 transcripts met the selection criteria of log_2_ ratio < −1.0-fold (*p* < 0.001; Supplementary Table S1, Figure S1). Of these, the downregulation of *EMP2* transcript significantly predicts inferior overall survival (*p* = 0.0385). Therefore, EMP2 might play a tumor suppressor role in UBUC.

### Alternations of EMP2 levels affected cell cycle distribution, cell viability, cell proliferation and colony formation via regulation of G_2_/M checkpoints in UBUC-derived cells

**Figure 1:**
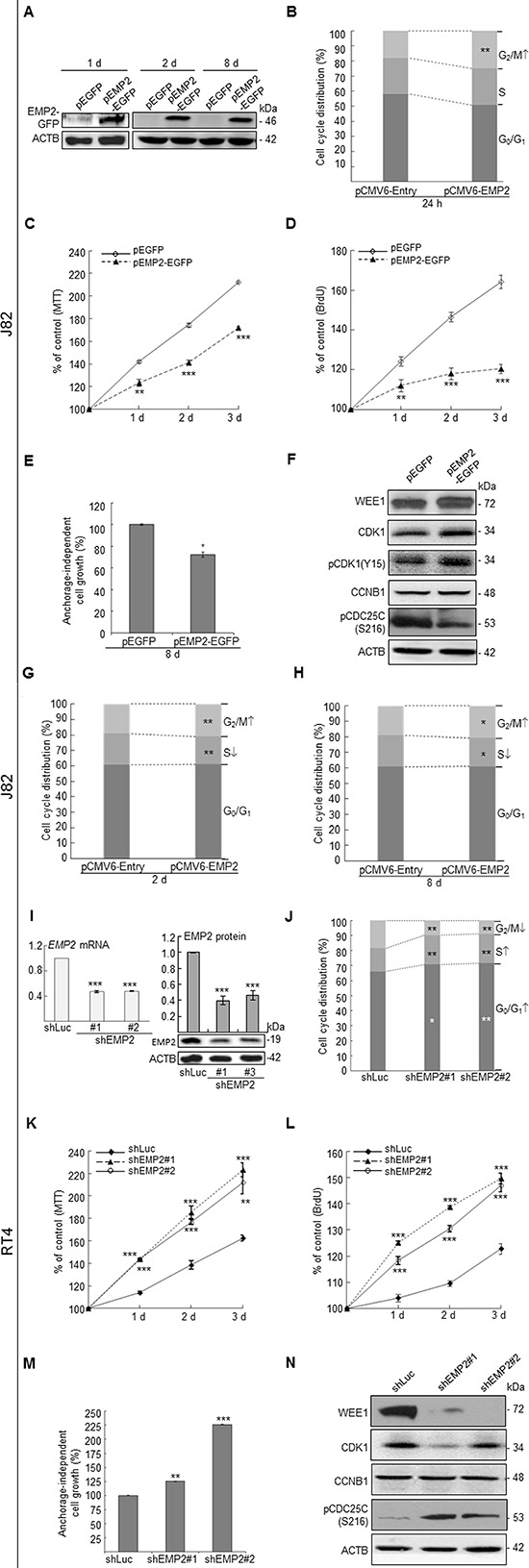
*In vitro* assay demonstrated that the *EMP2* gene playa a tumor suppressor role in UBUC-derived cells Immunoblotting, quantitative RT-PCR, flow cytometric, 3-(4,5-dimethylthiazol-2-yl)-2,5-diphenyltetrazolium bromide (MTT), 5-bromo-2′-deoxyuridine (BrdU) and soft agar assays along with transfection of pEMP2-EGFP in J82 cells exhibited that stably exogenous EMP2 expression induced **(A)** EMP2-GFP protein levels, **(B)** G_2_/M cell cycle arrest; however, suppressed **(C)** cell viability, **(D)** cell proliferation, and **(E)** anchorage-independent cell growth via upregulation of WEE1, CDK1, pCDK1(Y15) and downregulation of pCDC25C(S216) protein levels **(F)**. Stable overexpression of the EMP2 gene for 2 d **(G)** and 8 d **(H)** induced G_2_/M cell cycle arrest as well. On the other hand, stable knockdown of the *EMP2* gene in RT4 cells **(I)** suppressed *EMP2* mRNA and EMP2 protein levels, induced **(J)** cell cycle progression to G_0_/G_1_ and S phases, **(K)** cell viability, **(L)** cell proliferation, **(M)** anchorage-independent cell growth via downregulation of WEE1, CDK1 and upregulation of pCDC25C(S216) protein levels **(N)**. All experiments were triplicated and results are expressed as mean ± SEM. For immunoblotting analysis, one representative image is shown (A, F, I & N). Statistical significance: *, *p* < 0.05; **, *p* < 0.01 and ***, *p* < 0.001.

The *EMP2* mRNA and protein levels are notably higher expressed in HUC and RT4 than those in TSGH8301 and J82 cells (Supplementary Figure S2). Therefore, J82 and RT4 cells, respectively, were used for overexpression and knockdown of the *EMP2* gene for functional studies *in vitro*. Immunoblotting, flow cytometric, MTT, BrdU and soft agar colony formation (anchorage-independent cell growth) assays demonstrated that exogenous expression of *EMP2* in J82 cells stably expressed EMP2-GFP fusion protein, induced G_2_/M cell cycle arrest (*p* < 0.05), suppressed cell viability (*p* < 0.01), cell proliferation (*p* < 0.01) and colony formation/anchorage-independent cell growth (*p* < 0.05; see also Supplementary Figure S3A) via upregulation of WEE1 G2 checkpoint kinase (WEE1), cyclin-dependent kinase 1 (CDK1), CDK1(phospho-Y15) [pCDK1(Y15)] and downregulation of cell division cycle 25C(phospho-S216) [pCDC25C(S216)] (Figure 1A–1H). Conversely, as shown in Figure 1I–1N, stable knockdown of *EMP2* gene in RT4 cells inhibited *EMP2* mRNA (*p* < 0.001) and protein (*p* < 0.01) levels, induced cell cycle progression to G_0_/G_1_ (*p* < 0.05) and S (*p* < 0.01) phases, increased cell viability (*p* < 0.01), cell proliferation (*p* < 0.001) and colony formation/anchorage-independent cell growth (*p* < 0.01; see also Supplementary Figure S3B). These results suggested that *EMP2* suppresses cell proliferation and cell cycle progression through regulation of G_2_/M checkpoints in distinct UBUC-derived cells.

### Genistein upregulates cAMP responsive element binding protein 1 and subsequently transactivates *EMP2 in vitro*

**Figure 2:**
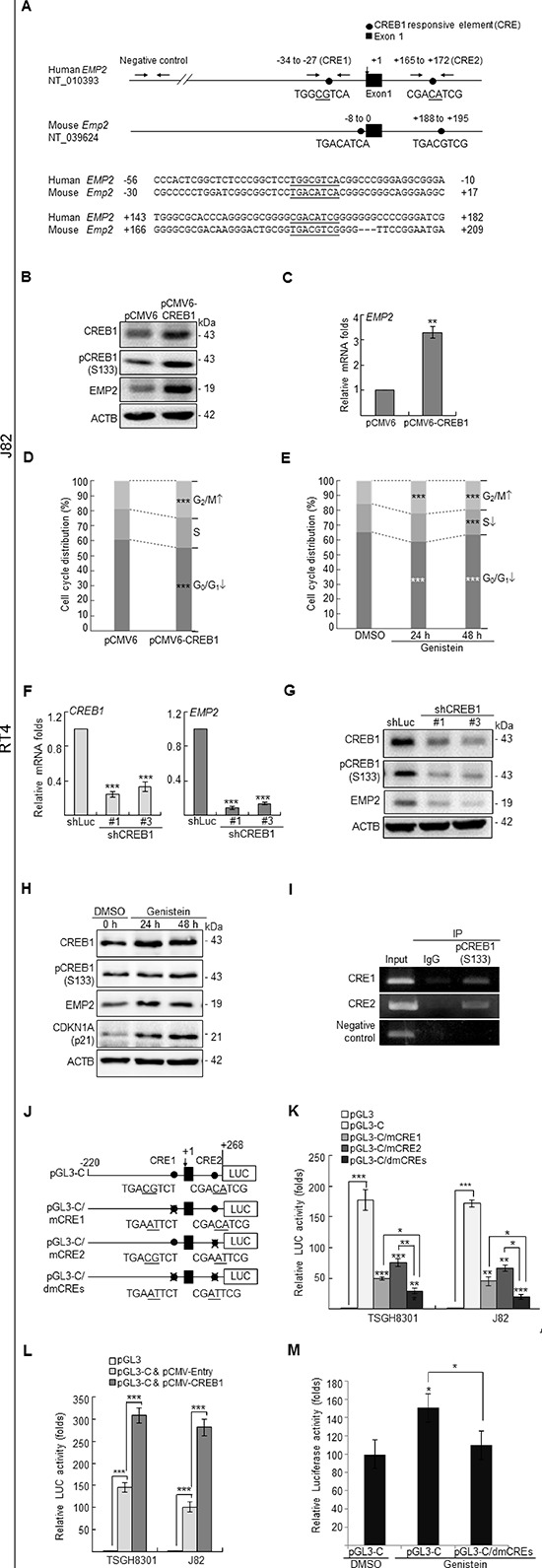
Genistein upregulated CREB1 and pCREB1(S133) protein levels, and pCREB1(Ser133) transactivates *EMP2* gene in UBUC-derived cells **(A)** Phylogenetic footprinting identified two conserved CREB1-responsive elements (CRE1 & CRE2) in the proximal promoter region of human *EMP2* and mouse *Emp2* orthologs, the first nucleotide of exon 1 was defined as +1. In J82 cells, transfection of the pCMV-CREB1 plasmid notably induced **(B)** CREB1, pCREB1(S133) and EMP2 protein, and **(C)** *EMP2* mRNA levels. **(D, E)** Both stably transfection pCMV-CREB1 plasmid and genistein treatments induced G_2_/M cell cycle arrest in J82 cells. **(F, G)** Conversely, stable transfection of shRNAi plasmids targeting *CREB1* gene suppressed *CREB1* and *EMP2* mRNA, and CREB1, pCREB1(S133) and EMP2 protein levels in RT4 cells. **(H)** In J82 cells, genistein treatments (10 μg/mL in DMSO) for 24 and 48 h notably induced CREB1, pCREB1(S133) and EMP2 protein levels. CDKN1A is a well-known target for genistein, was applied as a positive control. **(I)** Chromatin immunoprecipitation (IP) assay further confirmed that pCREB1(S133) protein interacts with both potential CREs; IgG was served as a negative control. **(J)** One DNA fragment (–220 to +268) containing two CREs of the *EMP2* proximal promoter region was cloned into pGL3 reporter vector, designated as pGL3-C. Site-directed mutagenesis (underlined) at CRE1 (pGL3-C/mCRE1), CRE2 (pGL3-C/mCRE2) and double mutagenesis at both CREs (pGL3-C/dmCREs) were also cloned into the pCL3 reporter vector. **(K)** In TGSH8301 and J82 cell lines with lower endogenous *EMP2* levels, dual luciferase assays demonstrated that transfection of pGL3-C increased promoter activities, compared to those transfections with the pGL3 control. However, the promoter activities were decreased after transfection of pGL3-C/mCRE1, pGL3-C/mCRE2 or pGL3-C/dmCREs plasmid for 24 h, compared to those transfections with the pGL3-C control. The promoter activity was further diminished after transfection of the pGL3-C/dmCREs plasmid, compared to those of transfection with either plasmid with single mutation, pGL3-C/mCRE1 or pGL3-C/mCRE2. **(L)** Transfection of pCMV-CREB1 for 24 h increased the pGL3-C promoter activity, compared to those of transfection with pCMV-Entry plasmid in both TSGH8301 and J82 cell lines. **(M)** In J82 cells, treatment with genistein (10 μg/mL in DMSO) increased the activity of pGL3-C, compared to the control (pGL3). However, genistein did not alter the promoter activity with double mutations in CREs (pGL3-C/dmCREs). All experiments were triplicated and results are expressed as mean ± SEM. For immunoblotting analysis, one representative image is shown (B, G, H). Statistical significance: *, *p* < 0.05; **, *p* < 0.01 and ***, *p* < 0.001.

To further identify any transcription factor that might regulate *EMP2* expression, phylogenetic footprinting was performed. Two putative cAMP responsive elements (CREs) in the *EMP2* proximal promoter region were identified, denoted as CRE1 and CRE2 (Figure 2A). Exogenous expression of cAMP responsive element binding protein 1 (*CREB1*) in J82 cells notably upregulated CREB1, pCREB1(S133), EMP2 protein and *EMP2* mRNA (*p* < 0.001) levels (Figure 2B, 2C). Stable overexpression of *CREB1* gene (*p* < 0.001) or genistein treatments (10 μg/mL) for 24 h (*p* < 0.001) and 48 h (*p* < 0.001) in J82 cells, significantly induced G_2_/M cell cycle arrest (Figure 2D, 2E). In contrast, stable knockdown of *CREB1* gene in RT4 cells downregulated *CREB1* (*p* < 0.001) and *EMP2* (*p* < 0.001) mRNA (Figure 2F); CREB1, pCREB1(S133) and EMP2 protein (Figure 2G) levels. Further, genistein treatments for 24 and 48 h notably induced CREB1, pCREB1(S133) and EMP2 protein abundance in J82 cells (Figure 2H). ChIP assay confirmed that pCREB1(S133) protein interacts with both CRE1 and CRE2 in the *EMP2* proximal promoter region, while IgG did not (Figure 2I). Single, double mutations at CRE1 and/or CRE2 were next created (Figure 2J), and a dual luciferase assay additionally demonstrated that the *EMP2* promoter activity decreased when either single mutation (pGL3-C/mCRE1 or pGL3-C/mCRE2) was introduced (*p* < 0.001), compared to those with pGL3-C plasmid (wild type). The promoter activity of *EMP2* gene was further diminished when double mutations (pGL3-C/dmCREs) were incorporated, compared to either single mutant (*p* < 0.05) (Figure 2K). Exogenous expression of the *CREB1* gene in both TSGH8301 and J82 cell lines, with low endogenous EMP2 levels, elevated pGL3-C activity (Figure 2L). Genistein increased pGL3-C activity (*p* < 0.05); however, it did not stimulate the promoter activity when double mutations were introduced (pGL3-C/dmCREs) in J82 cells (Figure 2M). Therefore, genistein induced *EMP2* transcription via upregulation of *CREB1* mRNA, CREB1 and pCREB1(S133) protein levels, as well as enhancement of the interaction between pCREB1(S133) and CREs on the *EMP2* proximal promoter region.

### Downregulation of EMP2 confers worse outcomes in UBUC patients

**Figure 3:**
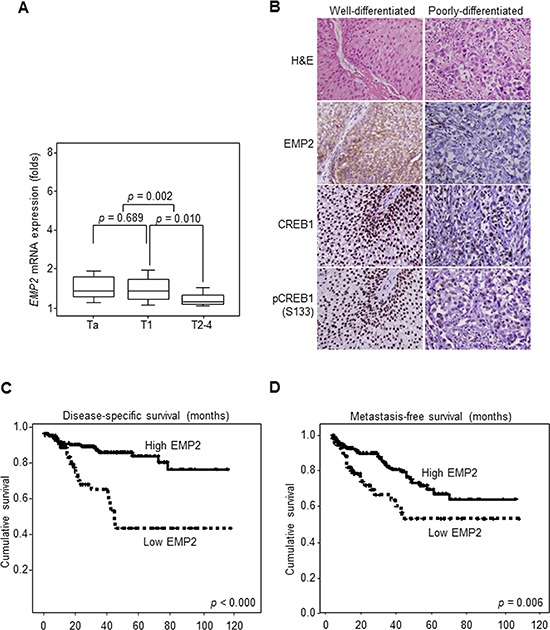
Downregulation of EMP2 immunointensity confers poor disease-specific survival (DSS) and metastasis-free survival (MFS) in UBUC patients **(A)** Quantitative RT-PCR demonstrated that *EMP2* mRNA levels were lower expressed in UBUC patients with high primary tumor stage (pT2-T4) than those with low stages (Ta-T1). **(B)** High EMP2 immunointensity in the representative poorly-differentiated, compared to that of well-differentiated UBUC specimen. Similarly, loss of CREB1 and pCREB1(S133) immunointensities were identified in poorly-differentiated tumors. **(C, D)** Kaplan-Meier curves plotted that high EMP2 protein level predicted superior DDS and MFS.

As shown in Figure 3A, *EMP2* mRNA was higher expressed in low-stage (Ta-T1) than high-stage (T2–4) UBUC patients (*p* = 0.002). Higher EMP2, CREB1 and pCREB1(S133) immunointensities were also identified in well-differentiated tumors, compared to those of poorly-differentiated ones (Figure 3B). Correlations between EMP2, CREB1 or pCREB1(S133) immunointensity and various clinicopathological factors are listed in Table 1. Univariate log-rank analysis identified that pT, nodal status, histological grade, vascular invasion, perineurial invasion, mitotic activity and EMP2 immunointensity were significantly correlated with disease-specific survival (DSS) and metastasis-free survival (MFS) in UBUC patients (Table 2). Kaplan-Meier plots revealed that low EMP2 immunointensity predicted worse DSS (*p* < 0.000) and MFS (*p* = 0.006) (Figure 3C, 3D). Multivariate analysis additionally demonstrated that pT and EMP2 immunointensity significantly correlate to DSS; pT, mitotic activity, and nodal status considerably correlated with MFS (Table 3).

**Table 1:** Correlation between EMP2, CREB1 and pCREB1(S133) expression level (labeling index: LI) and various clinicopathological factors

Parameters	Case	EMP2 LI	*p* value	CREB1 LI	*p* value	pCREB1(S133) LI	*p* value
Gender			0.551		0.889		0.722
Male	177	53.00 ± 4.24		95.31 ± 1.14		74.38 ± 2.72	
Female	65	55.20 ± 2.63		95.01 ± 0.68		70.45 ± 2.03	
Age (years)			0.607		0.325		0.459
< 60	70	53.43 ± 4.15		95.79 ± 0.62		70.36 ± 3.02	
≥ 60	172	55.09 ± 2.65		94.80 ± 0.78		71.98 ± 2.00	
Primary tumor (pT)			< 0.001^*^		0.392		< 0.001^*^
Ta	73	75.07 ± 3.19		96.64 ± 0.44		78.97 ± 2.35	
T1	73	56.58 ± 3.94		95.89 ± 0.80		78.29 ± 2.48	
T2-T4	96	37.55 ± 3.25		93.28 ± 1.29		60.68 ± 2.96	
Nodal status (N)			0.012^*^		0.101		0.031^*^
N0	220	56.41 ± 2.32		95.38 ± 0.56		72.75 ± 1.68	
N1-N2	22	38.59 ± 7.05		92.27 ± 3.12		59.09 ± 6.49	
Histological grade			< 0.001^*^		0.152		0.045^*^
Low	45	74.00 ± 3.90		97.22 ± 0.47		80.67 ± 2.18	
High	197	50.18 ± 2.49		94.59 ± 0.71		69.42 ± 1.94	
Vascular invasion			< 0.001^*^		0.027^*^		< 0.001^*^
Absent	202	58.51 ± 2.38		95.82 ± 0.53		74.98 ± 1.68	
Present	40	34.88 ± 5.13		91.38 ± 2.23		54.00 ± 4.63	
Perineurial invasion			0.083		0.059		0.141
Absent	225	55.47 ± 2.31		95.33 ± 0.59		70.09 ± 1.71	
Present	17	43.23 ± 8.35		91.76 ± 3.00		63.82 ± 6.48	
Mitotic activity (10 high power fields)	242	r = −0.343	< 0.001^*^	r = −0.008	0.888	r = −0.128	0.047^*^
Tumor necrosis			0.074		0.916		0.532
Absent	153	57.55 ± 2.77		95.03 ± 0.72		70.03 ± 2.19	
Present	89	49.55 ± 3.71		95.17 ± 1.01		74.04 ± 2.47	
EMP2 (LI)	242			r = 0.253	< 0.001^*^	r = 0.487	< 0.001^*^
pCREB1(S133) (LI)	242			r = 0.538	< 0.001^*^		

* statistically significant

**Table 2:** Univariate log-rank analyses

Parameters	Disease-specific survival			Metastasis-free survival	
	Case	Event	*p* value	Event	*p* value
Gender			0.9560		0.4970
Male	177	30		47	
Female	65	10		13	
Age (years)			0.2493		0.9305
< 60	70	9		18	
≥ 60	172	31		42	
Primary tumor (T)			< 0.0001^*^		< 0.0001^*^
Ta	73	1		4	
T1	73	8		18	
T2–4	96	31		38	
Nodal status (N)			0.0042^*^		< 0.0001^*^
N0	220	33		49	
N1–2	20	7		11	
Histological grade			0.0061^*^		0.0019^*^
Low	45	2		4	
High	197	38		56	
Vascular invasion			0.0170^*^		0.0012^*^
Absent	202	29		43	
Present	40	11		17	
Perineurial invasion			0.0219^*^		0.0041^*^
Absent	225	35		58	
Present	17	5		2	
Mitotic activity (10 high power fields)			0.0002^*^		0.0003^*^
Low (< 10)	113	10		19	
High (≥ 10)	129	30		41	
Tumor necrosis			0.6036		0.9119
Absent	153	25		39	
Present	89	15		21	
EMP2 Labeling index (LI)			< 0.0001^*^		0.0059^*^
Intact (2+–4+)	171	17		36	
Loss (0–1+)	71	23		24	
CREB LI			0.3198		0.0963
High (4+)	106	15		21	
Low (0–3+)	136	25		39	
pCREB1(S133) LI			0.0523		0.7274
High (4+)	106	27		27	
Low (0–3+)	136	13		33	

* Statistically significant

**Table 3:** Multivariate analysis for disease-specific and metastasis-free survivals

Parameter	Disease-specific survival			Metastasis-free survival		
	Hazard ratio	95% CI^1^	*P* value	Hazard ratio	95% CI^1^	*P* value
Primary tumor (pT)						
Ta	1	-	0.0054^*^	1	-	0.0123^*^
T1	7.246	0.724–72.533		4.258	1.148–15.790	
T2–4	19.622	2.072–182.055		5.632	1.534–20.679	
EMP2 labeling index		-				
High expression (> 20)	1	-	0.0492^*^	1	-	0.5982
Low expression (≤ 20)	1.970	1.002–3.870		1.190	0.684–2.071	
Mitotic activity						
Low (< 10 per 10 high power fields)	1	-	0.0528	1	-	0.0344^*^
High (≥ 10 per 10 high power fields)	2.197	0.990–4.872		1.754	0.965–3.188	
Vascular invasion						
Absent	1	-	0.3009	1	-	0.9845
Present	1.552	0.675–3.567		1.116	0.552–2.258	
Perineurial invasion						
Absent	1	-	0.3552	1	-	0.3483
Present	1.648	0.571–4.753		1.493	0.648–3.441	
Nodal status (N)						
N0	1	-	0.3661	1	-	0.0339^*^
N1–2	1.493	0.626–3.558		2.282	1.109–4.695	
Histological grade						
Low	1	-	0.7667	1	-	0.5668
High	1.289	0.241–6.913		0.966	0.269–3.464	

1 CI, confidence interval

* statistically significant

### Genistein inhibited tumor growth *in vivo*

**Figure 4:**
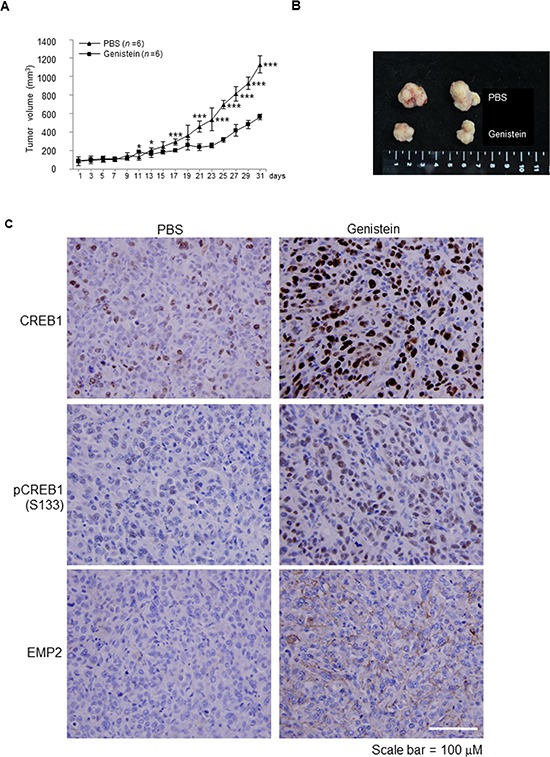
Genistein inhibited tumor growth in a NOD/SCID xenograft model J82 cells (1 × 10^7^) were mixed with Matrigel and injected into flank sites of mice (*n* = 12). After the cells grew for 30 days, tumors (~ 100 mm^3^) were directly injected with genistein (0.2 mg; *n* = 6) or PBS (control; *n* = 6) twice per week for 31 days. **(A)** Treatment with genistein for 19 days notably inhibited tumor growth, compared to the control (PBS). **(B)** After sacrifice, tumors were dissected from animals and two representative tumors from each group are shown. **(C)** Immunohistochemistry showed that genistein treatments noticeably upregulated CREB1, pCREB1(S133) and EMP2 protein levels *in vivo*. Statistical significance: *, *p* < 0.05 and ***, *p* < 0.001.

To elucidate whether genistein inhibits tumor growth *in vivo*, the mouse xenograft model was used. Approximately 30 day after cell injection, tumors grew into ~100 mm^3^. Treatment with genistein twice a week directly in tumors notably suppressed tumor growth (*p* < 0.001), compared to the PBS control group (Figure 4A, 4B), suggesting that genistein inhibited cell growth *in vivo*. Immunohistochemistry further showed that genistein noticeably induced CREB1, pCREB1(S133) and EMP2 protein levels in xenografts (Figure 4C).

### Knockdown of *EMP2* and/or *CREB1* enhanced tumor growth *in vivo*

**Figure 5:**
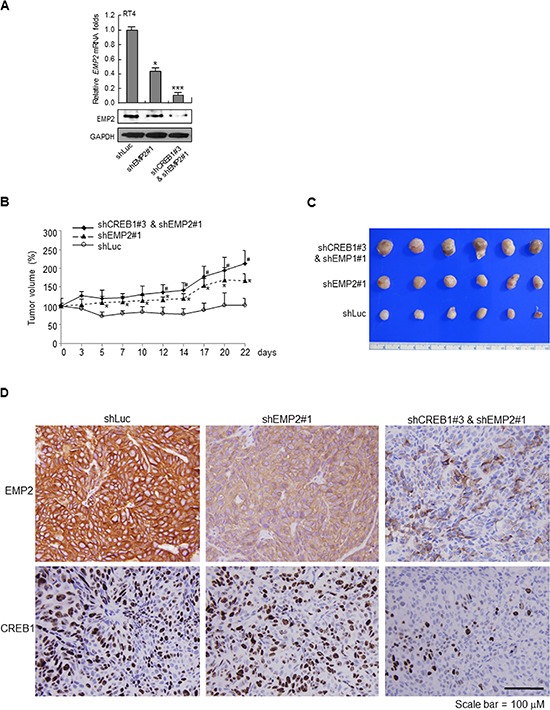
Stable knockdown of *EMP2* gene and double knockdown of *CREB1* and *EMP2* genes enhanced tumor growth in NOD/SCID xenograft models **(A)** The *EMP2* mRNA and protein levels were downregulated after stable transfection of shEMP#1 and cotransfection of shCREB1#3 and shEMP2# plasmids into RT4 cells. Knockdown cells (5 × 10^6^) were mixed with Matrigel and injected into flank sites of mice (*n* = 6 for each group). **(B)** Stable knockdown of *EMP2* gene (shEMP2#1) increased tumor growth, compared to the control group, shLuc (*, *p* < 0.05). Double knockdown of *CREB1* and *EMP2* genes (shCREB1#3 & shEMP2#1) further enhanced tumor growth, compared to the shEMP2#1 group (^#^, *p* < 0.05). **(C)** After sacrifice, tumors were dissected from animals and tumors from each group are shown. **(D)** Immunohistochemistry on xenograft tissues displayed that knockdown of *EMP2* gene notably downregulated EMP2 protein level, however, double knockdown of *CREB1* and *EMP2* genes markedly downregulated both CREB1 and EMP2 protein levels.

The mouse xenograft model was also used to evaluate whether knockdown of *EMP2* and double knockdown of *CREB1* and *EMP2* affected tumor growth *in vivo*. In RT4 cells, both stable knockdown of *EMP2* gene (shEMP2#1), and double knockdown of *CREB1* and *EMP2* genes (shCREB1#3 & shEMP2#1) inhibited *EMP2* mRNA (*p* < 0.05; *p* < 0.01) and protein levels, compared to the control group (Figure 5A). In NOD/SCID mice, xenografts with *EMP2* stable knocked down RT4 cells showed larger tumors, compared to the control group (*, *p* < 0.05). Double knockdown of *CREB1* and *EMP2* genes exhibited larger tumors, compared to the *EMP2* knockdown group (^#^, *p* < 0.05; Figure 5B, 5C). Immunohistochemistry further demonstrated that stable knockdown of *EMP2* gene suppressed EMP2 protein levels, compared to the controls (shLuc). Double knockdown of *CREB1* and *EMP2* genes downregulated CREB1 and EMP2 immunointensities, compared to knockdown of *EMP2* gene alone (Figure 5D).

## DISCUSSION

In this study, we found that a high EMP2 protein level could be an independent prognostic factor for DSS in UBUC patients, suggesting that loss of EMP2 expression plays a crucial role in the mortality of UBUC, similar to its role in nasopharyngeal carcinoma [24] and UTUC [12] observed in our earlier studies. Based on our unpublished cohort containing 60 UBUCs analyzed by Affymetrix^®^ Human SNP Assay 6.0, the *EMP2* locus is infrequently altered, suggesting the possibilities of epigenetic and/or transcriptional regulation of *EMP2* gene (Supplementary Figure S4). Loss of EMP2 expression is common and has prognostic significance for DSS and local recurrence-free survival in an NPC cohort of 124 patients. The EMP2 protein was expressed more highly in the cytoplasm and/or membrane of squamous metaplasia and non-keratinizing NPCs than in undifferentiated cells [24]. Membranous expression of EMP2 in urothelial cells of the ureter and EMP2 downregulation results in inferior cancer-related survivals (*n* = 171) [12]. Except for epithelial cells, suppression subtractive hybridization has isolated the mouse ortholog *Emp2*, which suppresses B cell lymphoma tumorigenicity via a functional tumor suppressor phenotype [25]. Conversely, EMP2 was identified as an early predictor of endometrial cancers with unfavorable outcome by activation of protein tyrosine kinase 2 (PTK2 or FAK) and v-src avian sarcoma viral oncogene homolog (SRC) [26, 27]. The above ambiguities strongly suggested that EMP2 might have distinct characteristics depending on cellular context. Because EMP2 is a transmembrane protein, its downregulation might decrease the interactions with other proteins, including the membranous and nonmembranous in epithelial cells, and subsequently result in tumor progression in UBUC.

*In vitro* studies, immunohistochemistry in tumor specimens, and correlation analyses in tissue specimens confirmed that genistein increased CREB1, pCREB1(S133) and EMP2 protein levels, and that the *EMP2* was transactivated by pCREB1(S133). CREB1 is a kinase-inducible transcription factor. The activation of CREB1-dependent gene expression is depicted by two-state model, i.e., CREB1 is thought to bind constitutively to CREs. Upon stimulation of S133 phosphorylation, CREB binding protein (CREBBP) is recruited. The activity of CREBBP histone-acetyl transferase next relaxes the local chromatin, allowing the latent affinity of the Q2 domain of CREB1 for TAF4 RNA polymerase II, TATA box binding protein-associated factor, 135 kDa (TAF4) to stimulate the deposition of the RNA polymerase II initiation complex and start transcription [28]. A number of kinase signaling cascades converge on the phosphorylation of S133 in CREB1 [29] and differences in kinase kinetic can result in unique patterns of gene expression [30]. It is known that activation of CREB1 turns on the transcription of more than 5000 target genes, including proto-oncogenes, FBJ murine osteosarcoma viral oncogene homolog (FOS) [31], cell cycle regulatory genes, cyclin A1 [32] and cyclin D2 [33], and other genes related to growth and survival [28, 34]. On the other hand, pCREB1(S133), phosphorylated by protein kinase, AMP-activated, alpha 1 catalytic subunit (PRKAA1), has the ability to bind the canonical CRE in the promoter region subsequently transactivate tumor protein p53 (TP53) in response to glucose deprivation [35]. Moreover, it has been found that an histone deacetylase (HDAC) inhibitor, valproic acid, caused an increase in transcription of a DNA damage recognition gene, the xeroderma pigmentosum, complementation group C (*XPC*) via increasing binding of both CREB1 and SP1 transcription factors in both HTB4 and HTB9 UBUC-derived cell lines [36]. An early study also reported that genistein upregulated CREB1 and pCREB1(S133) protein levels in MCF7 cells [37]. All these suggested that target genes of the CREB1 transcription factor might be broader than what we currently appreciate. Importantly, results from *in vitro* analyses were generally reflective of observations from data mining, clinical associations and xenograft mice. There was no significant correlation between CREB1 or pCREB1(S133) protein level and DSS or MFS was found, signifying that EMP2 abundance rather than CREB1 or pCREB1(S133), plays a predominant role in the inhibition of UBUC progression.

We also identified that overexpression of *EMP2* induced G_2_/M cell cycle arrest, decreased cell viability, proliferation and colony formation/anchorage-independent cell growth by distinct upregulation of WEE1, CDK1 and pCDK1(Y15), and downregulation of pCDC25C(S216) in J82 cells, and knowndown of *EMP2* gene in RT4 exhibited the opposite results. EMP2 induced G_2_/M cell cycle arrest from day 1 to day 8, suggesting its strong effects on cell cycle regulation. Indeed, orderly progression of cells through the cell cycle is orchestrated by the sequential interaction and activation of CDKs existing in complexes with their cyclin substrates [38]. Conserved from yeast to mammals, mitosis is controlled by maturation-promoting factor (the CDK1/CCNB1 complex) [39], which is present in low level during interphase and peaks during mitotic progression [40]. In addition to the activation by cyclins, CDK1 activity can be negatively regulated by phosphorylation of two inhibitory residues, Y14 and Y15. The tyrosine kinase, WEE1, phosphorylates CDK1 at Y15 [41]. Alternatively, membrane-associated tyrosine/threonine 1 protein (PKMYT1) is a dual-specificity kinase that can phosphorylate both sites [42, 43], with a propensity toward Y14 [44]. These inhibitory phosphorylations are removed by CDC25 phosphatases [45]. Humans possess three CDC25 isoforms (CDC25A, B and C), that are overlapping and have unique roles [46]. During interphase growth and under DNA damage or stress, CDC25C is prevented from entering the nucleus (inactive) owing to S216 phosphorylation and interaction with tyrosine 3-monooxygenase/tryptophan 5-monooxygenase activation protein, theta (YWHAQ or 14–3-3) [40, 47, 48]. These findings reinforce our observations.

Altogether, we demonstrate low EMP2 protein levels in a subset of UBUCs with aggressive behaviors. In distinct UBUC-derived cell lines, EMP2 expression induces G_2_/M cell cycle arrest via regulation of G_2_/M checkpoints, WEE1, pCDK(Y15) and pCDC25C(S216), and subsequently decreases cell viability, proliferation and colony formation/anchorage-independent cell growth. Clinical associations, *in vitro* indications and xenograft mice serve as strong evidence that genistein inhibited tumor growth by upregulating CREB1 and pCREB1(S133) protein levels. The *EMP2* gene is thereafter transactivated by pCREB(S133). Accordingly, downregulation of the EMP2 protein can be used as an adverse prognostic factor for inferior outcomes in UBUC patients.

## MATERIALS AND METHODS

### Data mining on the GEO database to identify downregulated transcripts in UBUCs

Data mining on the GEO database identified one dataset GSE31684, analysis on 93 UBUCs using GeneChip^®^ Human Genome U133 Plus 2.0 Array [49]. To computerize the expression level, raw CEL files were imported into the Nexus Expression 3 software (BioDicovery) as described earlier [50], except for functional profiles were performed by focusing on transcripts with biological process of *cell proliferation* (GO:0008283). Those transcripts with *p* < 0.001 and log_2_-transformed fold change of expression > ± 1.0 were selected as candidates. To further identify the most critical transcript(s) related to UBUC progression, all probes targeting candidates were analyzed for their impacts on overall survival.

### Cell culture and genistein treatment

Human normal urothelial cells (HUC; #4320, ScienCell Research) were obtained and cultured with recommended medium (#4321, ScienCell Research Laboratories) in the poly-L-lysine coated flask (2 μg/cm^2^). Human UBUC-derived cell lines, RT4, TSGH8301 (Food Industry Research and Development Institute, Hsinchu, Taiwan) and J82 (ATCC) were respectively maintained in McCoy's 5A modified, RPMI-1640 and DMEM, with 10% (v/v) fetal bovine serum (Biological Industries), appropriate nutrients and antibiotics in a humidified incubator with 5% CO_2_ at 37°C. All media were obtained from CORNING. RT4, TSGH8301 and J82 cells were characterized as stage Ta [51], Ta [52] and T3 [53], respectively. Genistein (10 μg/mL, Sigma-Aldrich) was prepared in DMSO.

### Quantitative RT-PCR

Quantitative RT-PCR assay was applied to quantify the expression levels of *EMP2* and cAMP responsive element binding protein 1 (*CREB1*) transcripts using predesigned TaqMan^®^ assay reagents [*EMP2*: Hs00171315_m1, 88 bp; *CREB1*: Hs00231713_m1, 75 bp; polymerase (RNA) II (DNA directed) polypeptide A (*POLR2A*): Hs00172187_m1, 61 bp, internal control), StepOnePlus™ Real-Time PCR System (Life Technologies) and ΔΔC_T_ calculation. Briefly, total RNAs were extracted with TRIzol^®^ reagent (Life Technologies) from cells and reverse-transcribed using the High Capacity cDNA Reverse Transcription kit (Life Technologies). The relative expression folds of *EMP2* and *CREB1* transcripts were given by 2^−ΔΔCT^, where ΔΔC_T_ = ΔC_T_ (_RT4_, _TSGH8301 or J82, tumor specimen or transfected cell lines_) – ΔC_T_ (_HUC, nontumor or control_); ΔC_T_ represented the C_T_ of *EMP2* or *CREB1* subtracted from the C_T_ of *POLR2A* for cell lines and/or tissue specimens. Only samples with C_T_ value < 28 for *POLR2A* were considered to meet acceptable RNA quality standards and included in the analyses.

### Immunoblotting analysis

Cell lysates were prepared with RadioImmuno Precipitation Assay buffer (Upstate). Lysates containing equal amounts of protein were separated by SDS-PAGE and electroblotted onto Immobilon™-P Transfer Membrane (Millipore). The filters were individually probed with specific primary antibody. Protein bands were detected by the Western Lightning Chemiluminescence Reagent Plus Kit (Perkin-Elmer Life Sciences) with horseradish peroxide labeled secondary antibody as suggested by the manufacturer and visualized on a VersaDoc Image System (Bio-Rad). The intensity of bands was quantified by densitometry and normalized to ACTB in each lane. Anti-human EMP2 (1:100, HPA014711, Sigma-Aldrich), anti-cyclin dependent kinase 1 (CDK1; 1:200, sc-54, Santa Cruz), anti-pCDK1(Y15) (1:250, BD Transduction Laboratories™), anti-WEE1 (1:1000, #4936, Cell Signaling), anti-CCNB1 (1:500, sc-245, Santa Cruz), anti-pCDC25C(S216) (1:1000, #4901, Cell Signaling) and anti-pCREB1(S133) (1:200, sc-7978, Santa Cruz) were used as primary antibodies for immunoblotting analysis and anti-ACTB (1:3000, Chemicon) was served as a loading control.

### Expression plasmids and stable transfection

Primer sets, 5′-CTGGAATTCATGTTGGTCCTTCT TGCTTTC-3′ and 5′-TCAAGCTTCTTTGCGCTTCCTC AGTATCAG-3′, embracing *Eco* RI and *Hind* III sites (underlined), were used to amplify the *EMP2* complete DNAs (NM_001424, NCBI). The PCR products were sequencing verified, gel purified, and subcloned into the pEGFP-N3 plasmid (#6080–1, Clontech) to generate the pEMP2-EGFP plasmid. The pCMV6-Entry (PS10001), pCMV6-EMP2 (RC201995) and pCMV6-CREB1 (RC210577) plasmids were obtained from OriGene Technologies. Cells (1 × 10^6^) were transfected with 2 μg of pEGFP-N3 (control), pEMP2-EGFP, pCMV or pCMV-CREB1 plasmid by mixing with 8 μL PolyJet™ reagent (SignaGen^®^ Laboratories). Transfectants were selected with media containing 800 μg/mL of G418 (AMRESCO) for 7 d, and maintained in media with 400 μg/mL of G418 for subsequent experiments.

### Lentivirus production and stable knockdown of the *EMP2* and *CREB1* genes

Small hairpin RNA interference (shRNAi) plasmids were inserted into the pLK0.1 vector downstream of the U6 promoter. Clones were obtained from the National RNAi Core Facility, Institute of Molecular Biology, Academia Sinica, Taipei, Taiwan. A total of 5 and 4 plasmids targeting *EMP2* and *CREB1* genes were preliminarily screened. The *EMP2* and *CREB1* mRNA levels could be effectively downregulated by only 2 and 2 clones, respectively. Plasmids shEMP2#1 (TRCN0000072386: 5′-CAACACGAATTGCACAGTCAT-3′), shEMP2#2 (TRCN0000072387: 5′-GTTTGTCCTAACCTCCAT CAT-3′), shCREB1#1 (TRCN0000011085: 5′-CAG TGGATAGTGTAACTGATT-3′) and shCREB1#3 (TRCN0000007309: 5′-GCAAACATTAACCATGACC AA-3) were used for knockdown of *EMP2* and *CREB1* genes and shLuc (TRCN0000072243:5′-CTTCGAAATGTCCGTTCGGTT-3)' was used as a negative control clone. For stable shRNAi, lentiviral particles were produced. Briefly, Phoenix-AMPHO cells (ATCC) were seeded in 6-cm tissue culture plate at a density of 3 × 10^6^ in 5 mL medium with 10% FBS, 100 IU/mL penicillin and 100 μg/mL streptomycin (Corning^®^) overnight. PolyJet™ (15 μL, #SL100688, SignaGen^®^ Laboratories) was used to transfect the plasmid mixture [psPAX2 (2.25 μg, Addgene), PMD2.G (0.25 μg, Addgene) and 2.5 μg of shLuc (control), shEMP2#1, shEMP2#2, shCREB1#1 and/or shCREB1#3 plasmids], and the medium was changed after a 16 h incubation. Medium was collected and filtered (0.22 μm) at 40 h and 64 h post-transfection, aliquots of 1 mL were stored at –80°C. RT4 cells (1 × 10^6^) were next infected with media containing lentiviral particles containing polybrene (8 μg/mL) and incubated for another 24 h at 37°C. Afterward, media containing 4 μg/mL puromycin (Sigma-Aldrich) were used to select positive cells for 7 d and subsequently maintained in media containing 2 μg/mL puromycin for further experiments.

### Cell-cycle, cell viability, proliferation and soft agar assays

Flow cytometric, 3-(4, 5-Dimethylthiazol-2-yl)-2, 5-diphenyltetrazolium bromide (MTT), bromodeoxyuridine (BrdU) and soft agar assays were used to determine alternations of cell cycle distribution, cell viability, cell proliferation and colony formation/anchorage-independent cell growth after exogenous expression or knockdown of *EMP2* and/or CREB1 genes *in vitro*. For cell cycle analysis, 1 × 10^6^ cells were collected, washed with ice-cold PBS, fixed with 70% ethanol and stored at –20°C after stable transfection of pCMV6-Entry, pCMV6-EMP2 or pCMV6-CREB1 plasmid, or infection with shEMP2#1, shEMP2#1, shCREB1#1, shCREB#3, shCREB1#3 and shEMP1#1, or shLuc lentiviral particles. Before analysis, fixed cells were washed with ice-cold PBS for three times and treatments with 200 μg/mL RNase A (#R6513, Sigma-Aldrich) and 20 μg/mL propidium iodide (#P4170, Sigma-Aldrich). A total of 10, 000 events were analyzed; cell cycle distribution was analyzed by a Beckman Coulter Epics XL Flow Cytometer and the Modfit LT™ software (BD Biosciences) [54].

To determine cell viability and proliferation upon alternation of EMP2 expression levels, 2 × 10^3^ and 3 × 10^3^ cells were seeded on 96-well microplates for MTT and BrdU assays, respectively. After removing the medium, 20 μL of MTT (5 mg/mL; Sigma-Aldrich) were added to each well and cells were incubated for another 4 h. At the end of incubation, the MTT solution was replaced by 100 μL of DMSO. On the other hand, BrdU Cell Proliferation Assay Kit (QIA58, Calbiochem) was used to perform cell proliferation test. BrdU label (1:2000 dilution) was incubated for 24 h. Plates were then washed, stained with anti-BrdU antibody, and peroxidase-conjugated goat anti-mouse IgG. 3, 3′, 5, 5′-tetramethylbenzidine substrate (0.1 mL in ethanol) was next added into the immunocomplex and the reaction was terminated via adding 100 μL of sulfuric acid (2.5 N). Absorbances were afterward measured at wavelengths of 570 and 490 nm for MTT and BrdU assays, correspondingly, using a Beckman Coulter PARADIGM™ Detection Platform. Percentages of viable cells (%) and proliferation rate (%) were calculated as 100 × [(OD_indicated time after transfection_ – OD_7d after transfection_)/OD_7d after transfection_]. All experiments were triplicated and results are expressed as mean ± SEM.

CytoSelect™ 96-well *in vitro* tumor sensitivity assay (soft agar colony formation, CBA-150–5, CELL BIOLABS, INC) was used to analyze whether stable expression and knockdown of *EMP2* affected anchorage-independent cell growth. Briefly, 50 μL/well (in a 96-well sterile flat-bottom microplate) of the Base Agar Matrix Layer was prepared by mixing 1.25 mL of 2X DMEM/20% FBS medium, 1 mL of sterile water, 0.25 mL of melted 10X CytoSelect™ Agar Matrix Solution. Cell Suspension/Agar Matrix Layer under sterile conditions (75 μL/well) was made by mixing 1.75 mL of 2X DMEM/20% FBS medium, 1.375 mL of CytoSelect™ Matrix Diluent, 0.375 mL of melted 10X CytoSelect™ Agar Matrix Solution and 0.25 mL of Cell Suspension (5 × 10^3^ cells), according to the manufactures' instructions. The incubation periods were 8 days for both *EMP2*-overexpressed J82 and *EMP2*-knocked down RT4 cells. MTT assay was used to quantitate the anchorage-independent growth.

### Chromatin immunoprecipitation

Cells were grown overnight in 100-mm dishes to ~60–70% confluency (5 × 10^6^), cross-linked with formaldehyde, harvested, and subsequently sonicated (SONICATOR^®^ 3000, LLC) to obtain soluble chromatin (~500 bp). After dilution, the chromatin solutions were incubated with 5 μg of anti-pCREB1(S133) antibody or rabbit IgG (5 μg, non-specific control, #N101, Calbiochem), and satiated on a rotating platform at 4ºC overnight. Immunocomplexes were recovered with preblocked protein A-Sepharose beads (Life technologies) at 65ºC for 4 h. Samples were next digested with proteinase K (Sigma-Aldrich) for 1 h at 45ºC and the DNA from samples was obtained by phenol/chloroform extraction and ethanol precipitation. Primers spanning –34 to –27 (CRE1: 5′-CAAAGCTGGCCACAGAGC-3′; 5′-CTCCCTCCACCCTCTAGGC-3′) and +165 to +172 (CRE2: 5′-AGCCCAGAGCTTCAAAACAG-3′; 5′-CTGCTCCCGGTCCAGTAAGT-3′), residing on the *EMP2* proximal promoter region and one negative control (5′-CTGCAGTGAGTCTGGGTTCA-3′; 5′-TGCT GAGGGCTTAGTGTGTG-3′) on ~33 Kb upstream of the *EMP2* gene were used for ChIP PCR assay with an annealing temperature of 60ºC. Resultant amplicons were separated on 2%, 0.5X Tris/borate/EDTA agarose gels, stained with EtB”Out” nucleic acids staining solution (#FYD007, Yeastern), visualized and photographed with UV light.

### Generation of reporter constructs and site-directed mutagenesis

Constructs with single- and double-mutant of CREs residing on the *EMP2* proximal promoter region were performed using the QuickChange^®^ Lightning Site-Directed Mutagenesis Kit (#210518, Agilent). All constructs were verified by sequencing. A plasmid (pGL3-C) containing 488 bp (–220 to +268) fragment of the *EMP2* proximal promoter linked to the luciferase reporter gene was initially cloned into pGL3 vector (Promega) using primers 5′-CCGCTCGAGAGCCTCCCTTTCTCCCTTTTC-3′ and 5′-CCCAAGCTTCCCGTTACTGTCACCAATT-3′, with *Xho* I and *Hind* III sites (underlined). The plasmid was next used as a template for site-directed mutagenesis (shading) on CRE1 (primers: 5′-GCTCTCCCGGCTCCTGGATTCACGGCCCG GGAG GC-3′; 5′-GCCTCCCGGGCCGTGAATCCAGGA GC CGGGAGAGC-3′), and CRE2 (primers: 5′-CCCAG GGC GCGGGGCGACATCGGGGGGGCCCCG-3′; 5′-CGGGGCCCCCCCGATGTCGCCCCGC GCCCTGG G-3′) to construct pGL3-C/mCRE1 and pGL3-C/mCRE2 plasmids, respectively. Next, pGL3-C/mCRE1 was used as the template to generate the plasmid comprising CRE1/CRE2 double-mutant (pGL3-C/dmCREs; primers: 5′-CAGGGCGCGGGGCGAATTCGGGGGG-3′; 5′-CCCC CCGAATTCGCCCCGCGCCCTG-3′).

### Patients and tumor materials

The institutional review board of Chi-Mei Medical Center approved retrospective retrieval of 242 primary UBUC with available tissue blocks (IRB10207–001), which underwent surgical treatment with curative intent between Jan. 1998 and May 2004. For immunohistochemical study and survival analysis, 242 consecutive patients with primary urinary bladder urothelial carcinoma were retrieved. These patients had received surgical resection with curative intent between 1998 and 2004; those who underwent palliative resection were excluded. Patients with confirmed or suspicion of lymph node metastasis received regional lymph node dissection. Cisplatin-based post-operative adjuvant chemotherapy was performed in those with pT3 or pT4 status or nodal involvement. The histological diagnosis of UBUC was confirmed in all cases based on the latest World Health Organization classification. Grading of histological was assigned based on Edmonson and Steiner's criteria, while tumor staging was determined according to the 7^th^ Edition of the American Joint Committee on Cancer system (AJCC). Medical charts were reviewed for each patient to ascertain the accuracy of other pertinent clinicopathological data. Follow-up information was available in all cases with a median period of 42 months (range 3–176 months).

### Immunohistochemistry

Immunohistochemical staining was performed on representative tissue sections cut from formalin-fixed, paraffin-embedded tissues at 3-μm thickness as our previous study [24] with a few modifications. Slides were deparaffinized with xylene, rehydrated with ethanol, heated by microwave for retrieval of antigen epitopes in a 10 mM citrate buffer (pH 6) for 7 min. Endogenous peroxidase was quenched by 3% H_2_O_2_. Slides were washed with Tris buffered saline for 15 min and then incubated with a primary monoclonal antibody against EMP2 (1:20; HPA014711, Sigma-Aldrich), CREB1 (1:40, sc-186, Santa Cruz) and pCREB1(S133) (1:50, sc-7978, Santa Cruz), for 1 h, followed by antibody detection using a ChemMate EnVision™ kit (K5001; DAKO, Glostrup). Two pathologists (CF Li and HY Huang) blinded to clinicopathological information and patient outcomes, independently interpreted the immunostainings. The immunointensity was scored based on the extent of moderately to strongly-stained tumor cells exhibiting combined membranous and cytosolic (EMP2) or nuclear [CREB and pCREB(S133)] staining, and labeled as 0+, < 5%; 1+, ≥ 5%, but < 25%; 2+, ≥ 25%, but < 50%; 3+, ≥ 50%, but < 75%; and 4+, ≥ 75%, respectively. A specimen showing EMP2 staining less than 1+ was regarded as loss of EMP2 expression. For CREB1 and pCREB1(S133), 4+ staining were regarded as high expression.

### Tumor xenograft and genistein treatment *in vivo*

Cells were implanted into 12 NOD/SCID mice by subcutaneous injection: 1 × 10^7^ J82 cells were resuspended in 100 μL PBS, mixed with 100 μL Matrigel (BD Biosciences) and introduced into the right flank of 7 week old, male mice. The tumor size reached ~100 mm^3^ about 30 days after implantation. Literally 2 μL of genistein (0.1 mg/μL in DMSO) dissolving in 198 μL of PBS (*n* = 6) or 2 μL of DMSO in 198 μL of PBS (control, *n* = 6) were injected into the tumor twice per week for 4.5 weeks. For xenograft with *EMP2* knockdown; *CREB1* and *EMP2* double knockdown experiments, literally 5 × 10^6^ RT4 cells that were stably transfected with shEMP2#1 or shCREB1#3 plus shEMP2#1 were subcutaneously injected for 22 days before sacrifice. Tumor diameters were measured with a digital caliper every other day and the tumor volume in mm^3^ was calculated as volume = π/6(width)^2^ × length.

### Statistics

All calculations were performed by SPSS 14.0 software. To determine the prognostic impact of selected transcripts identified in GSE31684, the deposited cases were subdivided into two clusters based on the expression level of each transcript, detected by a specific probe and computerized by *k*-means clustering (*k* = 2). The survival difference of the two clusters was next calculated by log-rank analysis and plotted by Kaplan-Meier method for overall survival. The association and comparison between various clinicopathological factors and EMP2, CREB1, pCREB1(S133) immunointensities were assessed by the Chi-square test. The endpoint analyzed for survival analysis was DSS and MFS. Student's *t*-test was used to examine the significance of difference in fold changes of mRNA and protein levels; percentages of cell cycle distribution, cell viability, proliferation and anchorage-independent cell growth. For all analyses, two-sided tests of significance were used and a *p* value of < 0.05 was considered to be statistically significant.

## SUPPLEMENTARY FIGURES AND TABLE


