# Supplementary material for: The cAMP responsive element binding protein 1 transactivates epithelial membrane protein 2, a potential tumor suppressor in the urinary bladder urothelial carcinoma
Source: Oncotarget. 2015 Apr 13;6(11):9220–39. doi: 10.18632/oncotarget.3312 (PMC4496213; doi:10.18632/oncotarget.3312)
Supplement: Supplementary file 1 [file oncotarget-06-9220-s001.pdf]

SUPPLEMENTARY FIGURES AND TABLE

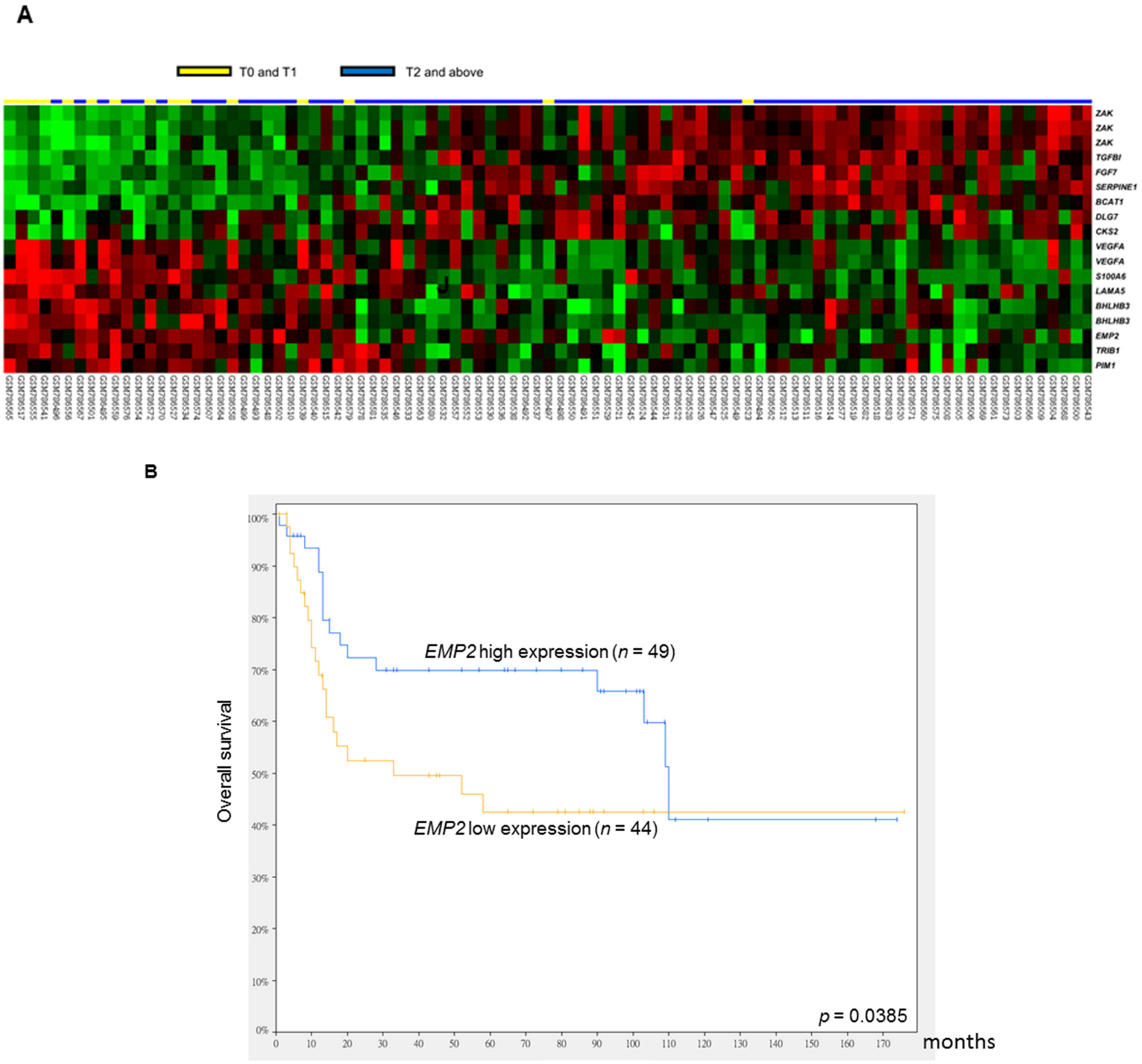

Supplementary Figure S1: Data mining on GSE31684 (GEO database) identified the expression levels of 14 transcripts were significantly altered in high (T2 and above), compared to low pT (Ta-T1) UBUCs, associated with biological process of cell proliferation (GO:0008283). The expression levels of these transcripts met the selection criteria of log2 ratio > ± 1.0-fold and ***p*** < 0.001. However, only the expression level of epithelial membrane protein 2 (*EMP2*) significantly predicts inferior overall survival. **(A)** A heat map of specimen identify with low (n = 15) and high (n = 73) pT are shown. Low expression values are green, progression to dark and reds for higher values. **(B)** Kaplan-Meier plots demonstrated that low *EMP2* mRNA level (k-means clustering, k = 2) predicts inferior overall survival ($p < 0.05$).

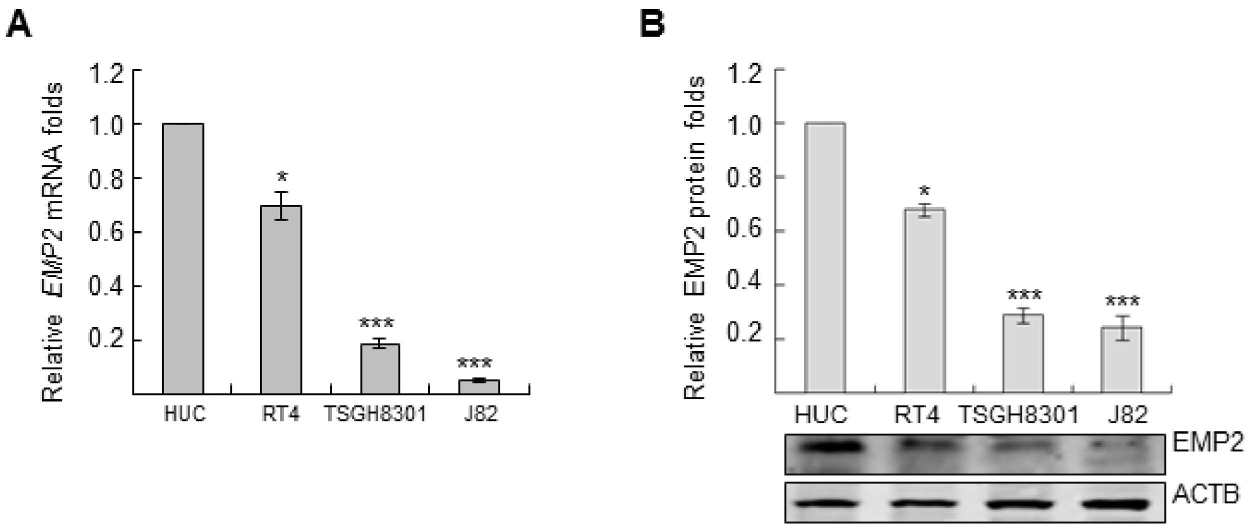

Supplementary Figure S2: **(A)** *EMP2* mRNA and **(B)** protein levels were higher expressed in normal human urothelial cells (HUC) and RT4 UBUC-derived cells, but lower expressed in TSGH8301 and J82 UBUC-derived cells.

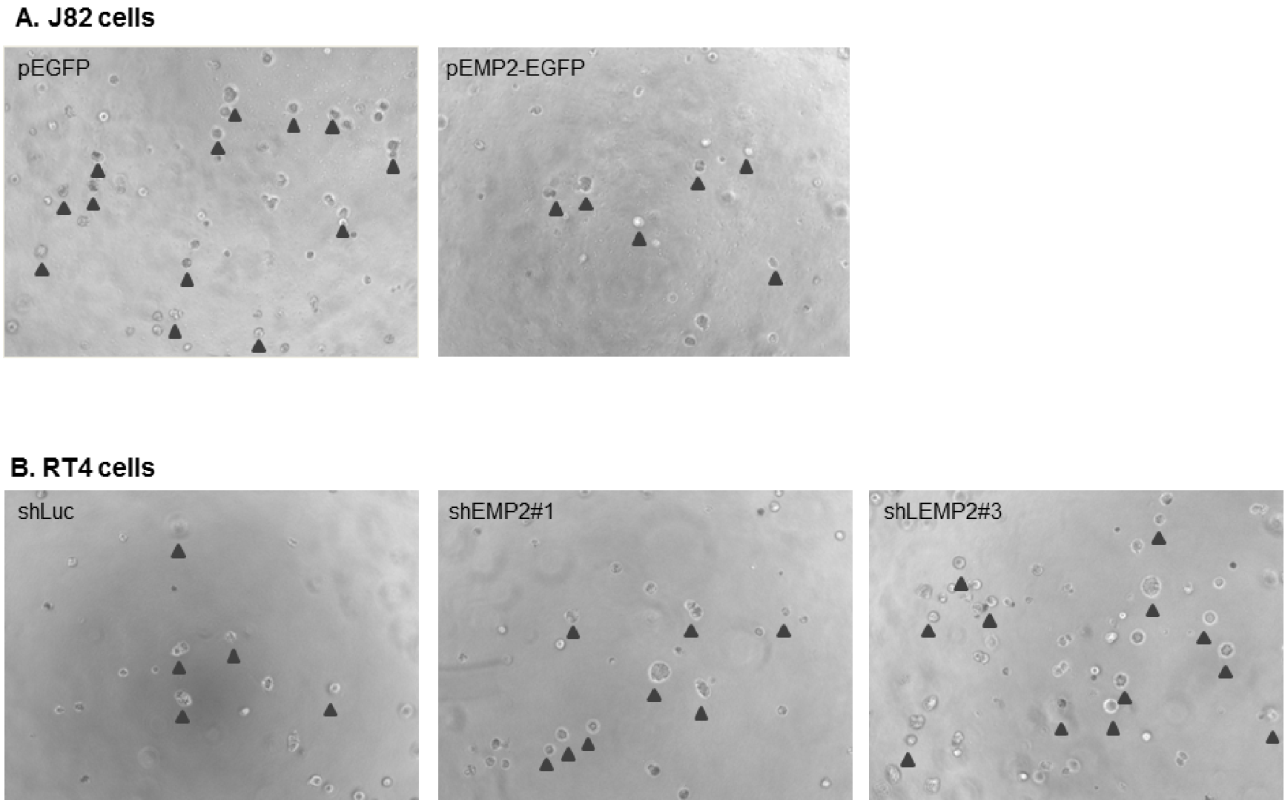

Supplementary Figure S3: Overexpression of *EMP2* suppressed, while knockdown induced anchorage-independent colony formation in UBUC-derived cells. J82 and RT4 cells (5×10^5) were stably transfected with pEMP2-EGFP and shEMP2 (shEMP2#1 & shEMP2#2) plasmids, respectively. Transfected cells (5×10^3) were then added in cell agar layer (CytoSelect™ 96-Well Transformation Assay, CELL BIOLABS, INC) and incubated for 8 d. **(A)** In J82 cells, exogenous EMP2 expression notably suppressed colony formation. **(B)** In RT4 cells, knockdown of *EMP2* gene markedly induced colony formation.

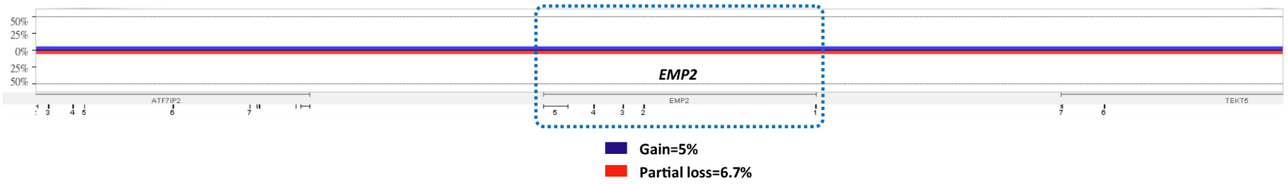

Supplementary Figure S4: Based on our unpublished cohort containing 60 UBUCs analyzed by Affymetrix® Human SNP Assay 6.0, the *EMP2* locus is infrequently altered, suggesting the possibilities of epigenetic and/or transcriptional regulation of *EMP2* gene.

Supplementary Table S1: Summary of downregulated transcripts associated with *cell proliferation* in the transcriptome of UBUC with higher primary tumor status

Gene symbol	Probe	Comparison log_2 ratio	Comparison *p* value	Gene name	Biological process
BCAT1	225285_at<br>226517_at	1.7653<br>1.8062	0.0015<br>0.0006	Branched chain aminotransferase 1; cytosolic	G_1/S transition of mitotic cell cycle, amino acid biosynthetic process, branched chain family amino acid biosynthetic process, branched chain family amino acid metabolic process, cell proliferation, metabolic process
BHLHB3	221530_s_at<br>223185_s_at	−1.5536<br>−1.2479	0.0007<br><0.0001	Basic helix-loop-helix domain containing; class B; 3	Cell differentiation, cell proliferation, organ morphogenesis, regulation of transcription, regulation of transcription; DNA-dependent, transcription
CDK6	224847_at	1.0795	0.0074	Cyclin-dependent kinase 6	G_1 phase of mitotic cell cycle, cell cycle, cell division, cell proliferation, protein amino acid phosphorylation, regulation of progression through cell cycle
CKS1B	201897_s_at	1.1312	0.0019	CDC28 protein kinase regulatory subunit 1B	Cell cycle, cell division, cell proliferation, regulation of cyclin-dependent protein kinase activity, regulation of progression through cell cycle
CKS2	204170_s_at	1.2649	0.0003	CDC28 protein kinase regulatory subunit 2	Cell cycle, cell division, cell proliferation, meiosis I, phosphoinositide-mediated signaling, regulation of cyclin-dependent protein kinase activity, spindle organization and biogenesis
DLG7	203764_at	1.2858	0.0003	Discs; large homolog 7 (Drosophila)	M phase of mitotic cell cycle, cell cycle, cell proliferation, cell-cell signaling, mitotic chromosome movement towards spindle pole, positive regulation of mitotic metaphase/anaphase transition

(*Continued*)

Gene symbol	Probe	Comparison log_2 ratio	Comparison *p* value	Gene name	Biological process
EMP2	204975_at<br>225078_at	−1.3533<br>−1.0881	0.0005<br>0.0031	Epithelial membrane protein 2	Cell death, cell proliferation, multicellular organismal development
FGF7	1554741_s_at	1.6435	0.0003	Fibroblast growth factor 7	Cell proliferation, cell-cell signaling, epidermis development, positive regulation of cell proliferation, regulation of progression through cell cycle, response to wounding, signal transduction
LAMA5	210150_s_at	–1.0435	0.0002	Laminin; alpha 5	Angiogenesis, cell adhesion, cell differentiation, cell migration, cell proliferation, cell recognition, cytoskeleton organization and biogenesis, embryonic development, endothelial cell differentiation, focal adhesion formation, integrin-mediated signaling pathway, regulation of cell adhesion, regulation of cell migration, regulation of embryonic development
PDGFC	218718_at	1.5657	0.001	Platelet derived growth factor C	Cell proliferation, central nervous system development, lipid metabolic process, regulation of progression through cell cycle
PIM1	209193_at	−1.1585	0.0002	Pim-1 oncogene	Cell proliferation, multicellular organismal development, negative regulation of apoptosis, protein amino acid phosphorylation
S100A6	228923_at	−2.4332	0	S100 calcium binding protein A6	Axonogenesis, cell cycle, cell proliferation, cell-cell signaling, positive regulation of fibroblast proliferation, regulation of progression through cell cycle, signal transduction
SERPINF1	202283_at	1.794	0.0007	Serpin peptidase inhibitor; clade F epithelium derived factor); member 1	Cell proliferation, multicellular organismal development, negative regulation of angiogenesis, positive regulation of neurogenesis
SPOCK1	202363_at	1.1471	0.01	Sparc/osteonectin; cwcv and kazal-like domains proteoglycan (testican) 1	Cell adhesion, cell motility, cell proliferation, multicellular organismal development, nervous system development
TGFBI	201506_at	1.9862	0.0007	Transforming growth factor; beta-induced; 68-kDa	Cell adhesion, cell proliferation, negative regulation of cell adhesion, response to stimulus, visual perception

(*Continued*)

Gene symbol	Probe	Comparison $\log_2$ ratio	Comparison *p* value	Gene name	Biological process
TRIB1	202241_at	−1.1839	0.0004	Tribbles homolog 1 (*Drosophila*)	Cell proliferation, protein amino acid phosphorylation, regulation of MAPK activity
VEGFA	211527_x_at<br>212171_x_at	–1.4201<br>−1.4173	0.0001<br>0	Vascular endothelial growth factor A	Angiogenesis, cell differentiation, cell migration, cell proliferation, induction of positive chemotaxis, multicellular organismal development, negative regulation of apoptosis, nervous system development, positive regulation of cell proliferation, positive regulation of vascular endothelial growth factor receptor signaling pathway, regulation of progression through cell cycle, response to hypoxia, signal transduction, vasculogenesis
ZAK	223519_at<br>225662_at<br>225665_at	1.3744<br>1.4198<br>1.5023	0.0001<br>0.0001<br>0	Sterile alpha motif and leucine zipper containing kinase AZK	DNA damage checkpoint, activation of JNK activity, activation of MAPKK activity, cell cycle, cell cycle arrest, cell cycle checkpoint, cell death, cell differentiation, cell proliferation, cytoskeleton organization and biogenesis, positive regulation of apoptosis, protein amino acid phosphorylation, protein kinase cascade, response to radiation, response to stress
ZEB1	212764_at	1.2668	0.0045	Zinc finger E-box binding homeobox 1	Cell proliferation, central nervous system development, embryonic morphogenesis, immune response, negative regulation of transcription from RNA polymerase II promoter, regulation of transcription, regulation of transcription from RNA polymerase II promoter, regulation of transcription; DNA-dependent, transcription
